# Evaluation of bacterial co-infections of the respiratory tract in COVID-19 patients admitted to ICU

**DOI:** 10.1186/s12879-020-05374-z

**Published:** 2020-09-01

**Authors:** Ehsan Sharifipour, Saeed Shams, Mohammad Esmkhani, Javad Khodadadi, Reza Fotouhi-Ardakani, Alireza Koohpaei, Zahra Doosti, Samad EJ Golzari

**Affiliations:** 1 Neuroscience Research Center, Qom University of Medical Sciences, Qom, Iran; 2 Cellular and Molecular Research Center, Faculty of Medicine, Pardis Campus, Qom University of Medical Sciences, Qom, Iran; 3 Ali Ebne Abitaleb Hospital, Qom, Iran; 4 Department of Infectious Diseases, Faculty of Medicine, Qom University of Medical Sciences, Qom, Iran; 5 Department of Medical Biotechnology, Faculty of Medicine, Qom University of Medical Sciences, Qom, Iran; 6 Occupational health & Safety Department, Faculty of Health, Qom University of Medical Sciences, Qom, Iran; 7 Department of Anaesthesiology and Intensive Care Medicine, Klinikum Dortmund, Dortmund, Germany

**Keywords:** 2019-nCoV, COVID-19, Co-infection, Bacteria, *A. baumannii*, *S. aureus*

## Abstract

**Background:**

COVID-19 is known as a new viral infection. Viral-bacterial co-infections are one of the biggest medical concerns, resulting in increased mortality rates. To date, few studies have investigated bacterial superinfections in COVID-19 patients. Hence, we designed the current study on COVID-19 patients admitted to ICUs.

**Methods:**

Nineteen patients admitted to our ICUs were enrolled in this study. To detect COVID-19, reverse transcription real-time polymerase chain reaction was performed. Endotracheal aspirate samples were also collected and cultured on different media to support the growth of the bacteria. After incubation, formed colonies on the media were identified using Gram staining and other biochemical tests. Antimicrobial susceptibility testing was carried out based on the CLSI recommendations.

**Results:**

Of nineteen COVID-19 patients, 11 (58%) patients were male and 8 (42%) were female, with a mean age of ~ 67 years old. The average ICU length of stay was ~ 15 days and at the end of the study, 18 cases (95%) expired and only was 1 case (5%) discharged. In total, all patients were found positive for bacterial infections, including seventeen *Acinetobacter baumannii* (90%) and two *Staphylococcus aureus* (10%) strains. There was no difference in the bacteria species detected in any of the sampling points. Seventeen of 17 strains of *Acinetobacter baumannii* were resistant to the evaluated antibiotics. No metallo-beta-lactamases -producing *Acinetobacter baumannii* strain was found. One of the *Staphylococcus aureus* isolates was detected as methicillin-resistant *Staphylococcus aureus* and isolated from the patient who died, while another *Staphylococcus aureus* strain was susceptible to tested drugs and identified as methicillin-sensitive *Staphylococcus aureus*.

**Conclusions:**

Our findings emphasize the concern of superinfection in COVID-19 patients due to *Acinetobacter baumannii* and *Staphylococcus aureus*. Consequently, it is important to pay attention to bacterial co-infections in critical patients positive for COVID-19.

## Background

A novel coronavirus known as severe acute respiratory syndrome coronavirus 2 (SARS-CoV-2, also called COVID-19 and 2019-nCoV) was first reported in Wuhan, Hubei Province, China in December 2019. Ever since the virus has been spreading worldwide claiming thousands of lives. Due to serious respiratory disease in humans, some patients need to be hospitalized and in severe cases intensive care with mechanical ventilation support is essential (~ 5–15%) [1, 2].

Although COVID-19 associated deaths have mainly occurred in the elderly with serious underlying diseases [3], nosocomial pneumonia (NP) in intensive care units remains a major risk factor for the patients and the health of patients, especially when intubated, may deteriorate in the presence of lower respiratory tract infections. Nosocomial infections (NIs) are usually described as infections acquired during hospitalization within 48–72 h after admission and they mainly spread through person-to-person contact, devices, and instruments [4]. Among microorganisms, the bacteria including *Staphylococcus* spp., *Enterococcus* spp., *Klebsiella pneumoniae*, *Enterobacter* spp., *Escherichia coli*, *Acinetobacter* spp., and *Pseudomonas* spp. are the most frequently detected causative agents of NIs [5].

These opportunistic pathogens can also cause superinfections, especially in combination with viral respiratory tract infections in hospitalized patients. However, even patients without underlying diseases and in all age groups may be at the risk of co-infections as well [6, 7].

Some studies have shown that viral agents such as influenza viruses can be associated with secondary bacterial pneumonia that might occur throughout hospitalization and lead to the death of individuals with or without preexisting respiratory diseases [8]. The damage of ciliated cells can also be observed in association with respiratory syncytial virus infection; it can result in deterioration of mucociliary clearance, increased adhesion of bacteria to mucins and, enhanced colonization of the bacteria in the airway. Moreover, new receptors for bacterial adherence can emerge following the virus-induced death of the airway epithelial cells [9]. In addition, after an acute inflammatory reaction and pulmonary tissue damage induced by viral infections, a resolving/repair phase of the lung tissue takes place. Due to varied immune responses in different individuals, this phase may cause an enhanced susceptibility to respiratory bacterial infections. Thus, bacterial superinfection can occur after a viral infection, which in turn might lead to increased morbidity and mortality [10].

Nevertheless, any probable contribution of the bacteria to the development of the infectious diseases caused by the newly discovered coronavirus is still completely unknown. In a study by Póvoa et al., the risk of ventilator-associated bacterial pneumonia in COVID-19 patients was studied [11]. In addition, although there are a few recently published retrospective reports of co-infections in patients with COVID-19 [12, 13], our study is adding to a growing evidence base of the role bacterial co-infections may have in COVID-19 patients. Therefore, our aim was to evaluate secondary bacterial infections and their antibiotic resistance in COVID-19 positive patients admitted to ICUs in Qom, the first city in Iran to report COVID-19 disease [14].

## Methods

### Patients

Nineteen critically ill patients admitted to the ICU wards in two referral hospitals for coronavirus in Qom, Iran, were enrolled in the present study. Patients were given antibiotics such as ceftriaxone and azithromycin before admission to the ICUs. Inclusion criteria were being infected by COVID-19, hospitalized, intubated, and mechanically ventilated > 48 h in ICUs. Ventilator-associated pneumonia (VAP) was identified based on the following criteria: a new and persistent (> 48 h) or progressive infiltrate on the chest radiograph plus 2 of the following minor criteria: fever > 38 °C or hypothermia < 36 °C, blood leukocyte count of > 10,000 cells/ml or < 5000 cells/ml, purulent tracheal secretions, or decrease in the PaO_2_/FiO_2_. In cases with clinically suspected pneumonia, VAP diagnosis was established with a positive quantitative culture (cut-off point ≥10^6^ [colony-forming units (CFU)/mL]) [15, 16]. All patients in our study were also neutropenic with elevated erythrocyte sedimentation rate and C-reactive protein (CRP); and had a history of sore throat, cough, and shortness of breath. To determine the status of the patients, i.e. death or discharge, we waited until the end of the admission of the last patient in our ICUs.

### Reverse transcription real-time polymerase chain reaction (RT-PCR) for the detection of COVID-19

This step was carried out once for each patient. Briefly, naso-pharyngeal samples were obtained using a specific swab (Medical Wire, UK) and then placed in a separate collection tube containing two ml of viral transport medium and immediately sent to the coronavirus-reference laboratory of the university. First, the extraction of the viral RNA was performed using a commercial kit according to the manufacturer’s protocol (GeneAll, Seoul, South Korea). Next, RT-PCR was performed using LightMix® Modular SARS and Wuhan CoV E-gene kit and using one-step RT-PCR polymerase Mix (Tib-Molbiol, Berlin, Germany) [17].

### Collection of the sample for the detection of bacterial superinfection

The collection of the samples for bacterial infections was repeated at four stages with an interval of ~ 3 days for each patient who still stayed in ICUs. Endotracheal aspirate (ETA) specimens were collected in sterile tubes based on a standard clinical protocol [18]. The specimens were immediately transferred to the bacteriological laboratory and were evaluated by conventional methods. First, the samples were cultured on Blood Agar, Chocolate Agar, Eosin Methylene Blue (EMB), and MacConkey Agar and then incubated at 37 °C for 24–72 h under standard conditions. The colonial growth of the bacteria was confirmed by Gram staining and other media and standard biochemical testing including (e.g., catalase, oxidase, Mannitol Salt Agar, Dnase, sensitive to some antibiotic disks, Triple Sugar Iron Agar [TSI], Sulfide Indole Motility [SIM], Methyl Red [MR]/Voges-Proskauer [VP], citrate, urease, etc.) (all media were acquired from Merck, Germany).

### Antimicrobial susceptibility testing (AST)

AST was separately performed on isolated bacteria at each stage of sampling and was evaluated by the standard disc diffusion method in accord with the recommendations of the Clinical & Laboratory Standards Institute or CLSI. The antimicrobial disks used were cefepime (CPM, 30 μg), cefoxitin (FOX, 30 μg), imipenem (IMP, 10 μg), ceftazidime (CAZ, 30 μg), amikacin (AK, 30 μg), meropenem (MEN, 10 μg), azithromycin (AZM, 15 μg), gentamicin (GM, 10 μg), tetracycline (TE, 30 μg), ceftriaxone (CRO, 30 μg), ciprofloxacin (CP, 5 μg), trimethoprim/sulfamethoxazole (SXT, 1.25/23.75 μg), levofloxacin (LEV 5 μg), penicillin (P, 10 U), erythromycin (E, 15 μg), piperacillin-tazobactam (PIT, 100/10 μg), ampicillin-sulbactam (AMS, 10/10 μg), and cefotaxime (CTX, 30 μg) (Padtan Teb, Iran). Minimum inhibitor concentration (MIC) was also performed for colistin and vancomycin according to CLSI protocol [19]. *S. aureus* ATCC 25923 and *E. coli* ATCC 25922 were used as standard strains.

### Phenotypical detection of metallo-beta-lactamase (MBL)

Phenotypic detection of MBL-producing clinical isolates was evaluated by the combination disk diffusion test (CDDT) and modified Hodge test (MHT) as previously described by Lee et al. [20].

## Results

All 19 patients were positive for bacterial infections. Out of 19 patients, 11 (58%) patients were male and 8 (42%) were female, with a mean ± standard deviation (SD) age of 67.1 ± 14.6 years (range of age 38–92 years). Of all patients, 16 cases (84%) had underlying diseases such as kidney disease, diabetes, hypertension, or heart diseases.

At the end of the study, 18 cases (95%) were dead (68.7 ± 13.3 years old), 89% (16 cases) of whom had underlying diseases, and 1 case (5%) was discharged (39 years old). At first collection, a total of 19 clinical specimens, all patients (100%) were found positive for bacterial infections, 17 cases (90%) for *Acinetobacter* (*A.*) *baumannii* and 2 cases (10%) for *Staphylococcus* (*S.*) *aureus*. In the stage of the 2, 3, and 4 of sampling, 15, 6, and 3 patients were included, respectively. In all samplings, the bacterium isolated from each patient remained the same. More information is presented in Table 1.

**Table 1:** More details about included patients

Patient	Stages of sampling (No.)	Infection	Length of stay in ICU (Days)	Underlying disease	Survived/ died
1	1, 2, 3, 4	*A. baumannii*	25	Diabetes and hypertension	Died
2	1, 2, 3, 4	*A. baumannii*	23	Diabetes and hypertension	Died
3	1, 2, 3, 4	*A. baumannii*	14	Diabetes	Died
4	1, 2, 3	*A. baumannii*	39	Diabetes, hypertension, and kidney disease	Died
5	1, 2, 3	*A. baumannii*	20	–	Died
6	1, 2, 3	*A. baumannii*	13	Diabetes and coronary artery disease	Died
7	1, 2	*A. baumannii*	27	Diabetes	Died
8	1, 2	*A. baumannii*	17	Diabetes	Died
9	1, 2	*A. baumannii*	16	Diabetes	Died
10	1, 2	MSSA	13	–	Survived
11	1, 2	*A. baumannii*	12	–	Died
12	1, 2	*A. baumannii*	11	Diabetes	Died
13	1, 2	*A. baumannii*	11	Diabetes and heart disease	Died
14	1, 2	*A. baumannii*	10	Diabetes and hypertension	Died
15	1, 2	*A. baumannii*	9	Diabetes	Died
16	1	*A. baumannii*	10	Diabetes and heart disease	Died
17	1	*A. baumannii*	9	Diabetes	Died
18	1	MRSA	9	Diabetes	Died
19	1	*A. baumannii*	2	Diabetes, hypertension, and coronary artery disease	Died

Overall, the mean ± SD length of pre-ICUs stay for all included patients was 3.8 ± 4.6 days, while the average in our ICUs was 15.3 ± 8.5 days (15.4 ± 8.7 and 13 days for expired and discharged patients, respectively). The median ICU length of stay for *A. baumannii* -positive and *S. aureus* -positive patients was 15.8 ± 8.8 and 11 ± 2.9 days, respectively.

Results of the antimicrobial susceptibility testing showed a high-level resistance of *A. baumannii* isolates to all tested antibiotics, except colistin with a resistance rate of 52%. No isolated *A. baumannii* strain produced MBLs and the resistance pattern of *A. baumannii* isolates was not different between expired and discharged patients.

One of the *S. aureus* isolates was detected as methicillin-resistant *Staphylococcus aureus* (MRSA) and resistant to all other evaluated agents i.e. penicillin, cefoxitin, azithromycin, erythromycin, gentamycin, co-trimoxazole, linezolid, and ciprofloxacin. No resistance was observed to vancomycin or tetracycline. The MRSA strain was isolated from a patient who expired on the 9th day of ICU admission. Another *S. aureus* strain, isolated from a discharged patient, was identified as methicillin-sensitive *Staphylococcus aureus* (MSSA) and susceptible to all the above-mentioned drugs.

Three of our patients (16%) had no underlying diseases. One of them was infected with the MSSA and the other two cases were infected with the *A. baumannii* strains. Among, only MSSA-infected patient was discharged and other two *A. baumannii*-infected patients were expired.

## Discussion

COVID-19, a viral pneumonia with an unusual outbreak, is considered as a new public health concern threatening us worldwide. Recent studies show that 2019-nCoV or SARS-CoV-2 originated from an animal source and later adapted to other variants as it crossed the species barrier to ultimately infect humans [21, 22]. In recent months, less attention has been paid to hospital-acquired infections and opportunistic microorganisms, which could be due to the outbreak of COVID-19 and its consequent long-term hospitalization of patients, and high workload on the healthcare personnel.

In this study, with a focus on secondary infection of the lower respiratory tract of patients*, A. baumannii* was the most common organism followed by *S. aureus*. In recent years, emerging strains of both species that have acquired additional genetic features have shown to be commonly associated with hypervirulence and resistant to many types of antibiotics [23, 24]. According to our infection control committee and laboratory reports, these were associated with other bacteria including *Pseudomonas aeruginosa, Escherichia coli, Klebsiella pneumoniae, Enterobacter spp., Serratia marcescens, and Citrobacter freundii,* etc. that were previously isolated from the ICU wards and non COVID-19 patients admitted to our ICUs. In addition, both *A. baumannii* and *S. aureus* were among the most isolated bacteria from non COVID-19 ICU patients in Iran and other countries [25]. In a 2019 study conducted in Tehran, Iran, *Klebsiella pneumoniae* and *Acinetobacter* had the highest rates of incidence in ICUs [26]. In a 2018 study, *A. baumannii* and *Klebsiella* spp. were the most common organism isolated in Mysuru, India [27]. In 2014, the most common ICU-acquired strains were *Acinetobacter baumannii*, *Pseudomonas aeruginosa, Stenotrophomonas maltophilia, Staphylococcus aureus, Enterococcus* spp., and *Klebsiella pneumonia* in Shanghai, China [28].

In the present study, our first samplings were performed in the patients who were admitted to ICUs for ≥9 days, except for 1 case with 2 days of admission. Certainly, this duration was an excellent opportunity for bacteria to infect the patients, and thus all of our first cultures were positive with secondary infection (19/19, 100%). This incidence rate is higher than similar recently published articles. In Fu et al. study, 13.9% (5 of 36) of the patients in the ICU were diagnosed with severe acute respiratory syndrome coronavirus 2 and secondary bacterial infection. In another report that was published from a UK secondary care setting, amongst 836 patients identified as SARS-CoV-2, 27 cases (3.2%) had early confirmed bacterial isolates identified (0–5 days post admission) rising to 51 cases (6.1%) during the admission [12, 13].

In addition, our result indicates a higher incidence than other published studies on non COVID-19 patients. In a study conducted in Shiraz, Iran, in 2009, Hassanzadeh et al. suggested that ICU-acquired infections were documented in 51.7% of ICU patients, with a mortality rate of 10.9% (5 patients) [29]. One of the reasons for the increase in infection rate in our study can be due to the simultaneous infection of the virus and bacterium. As previously mentioned, viruses can facilitate the attachment and colonization of the bacteria in the respiratory tract, which is certainly no exception for COVID-19; however, understanding the accurate mechanisms of interactions between novel coronavirus and other bacteria requires further research. Nevertheless, other factors such as ICU type, used equipment rate, admission/ discharge criteria, high workload/nurse ratio, etc. can also affect the quality of care and the rate of ICU acquired nosocomial infection [30, 31], especially in pandemics.

Except for colistin, *A. baumannii* strains showed widespread resistance to all different classes of antibiotics and no inhibition zone was observed in the disk diffusion method. Resistant isolates of the bacteria, especially *A. baumannii*, are not uncommon among admitted patients in the hospitals and hospital-acquired infections have become a major concern to health systems. Wang et al. showed that the resistance rate of *A. baumannii* isolates was approximately > 98% to piperacillin, imipenem, ceftriaxone, ciprofloxacin, and ceftazidime [32]. Castilho et al. also reported that *A. baumannii* isolates from ICUs in Goiânia, Brazil, were classified as multi-drug resistant (MDR) with a high incidence of resistance to carbapenems. The development of resistance to carbapenems and other *β*-lactams may be due to the production of the MBLs. These are one of the most common participating in resistance mechanisms that can inactivate a wide range of *β*-lactam antibiotics [33]. Nevertheless, no MBL-producing *A. baumannii* strain was isolated. However, the bacteria may use other strategies to resist the effects of antibiotics [34, 35].

In our study, one of the strains of *S. aureus* was identified as MRSA. This organism plays an important role in the severe complication of infections in ICU environments. The probability of acquiring MRSA may increase (> 2.5–4 times) in patients with longer stays in the ward, i.e. more than one week [36]. Different studies have also shown that lower respiratory tract infections caused by MRSA can be associated with a significant level of mortality in the patients admitted to ICUs [37, 38].

Due to the COVID-19 crisis conditions, we were not able to carry out MIC and other phenotypic confirmatory tests for evaluating extended-spectrum beta-lactamases or ESBLs, etc., as well as molecular assays for detecting resistance genes. Nevertheless, these pathogens showed extremely high rates of resistance to the majority of the antibacterial agents tested. This could not only delay the process of treatment and recovery of COVID-19 patients but also increase the mortality rate.

Based on our local strategies, all patients in the current study routinely received ceftriaxone and azithromycin (except for some contraindications or interactions) before admission to the ICUs. In the cases of the infection in ICU, these were changed to extended-spectrum antibiotics such as meropenem and vancomycin, but no changes in the isolation of our resistant bacteria were observed at different stages of sampling. However, the treatment protocols have been changed by the ICU medical team based on the obtained results of the cultures and the pattern of antibiotic resistance, e.g. in this study, combination therapy with meropenem, colistin, and ampicillin-sulbactam was used for the treatment of infections caused by the resistant strains of *Acinetobacter*.

Among our patients, three cases had no underlying diseases. One patient, infected by a susceptible strain of Staphylococci, was discharged, while two other patients, infected with multidrug-resistant *A. baumannii*, expired. Due to some limitations, the sample size of the current study was not sufficient for comparing and accurate statistical evaluation. However, further work is required to investigate whether there are increased mortality rates associated with patients co-infected with COVID-19 and antibiotic-resistant bacteria.

The median length of ICU stay among patients in our study was higher, 15 days (interquartile range, 2 to 39), compared with Zhou et al. study, which reported a length of stay of 8.0 days (4.0–12.0) of all patients with COVID-19 admitted to their ICU. Moreover, no bacterial pathogens were detected in their patients on admission [39]. It seems that the length of ICU stay can be prolonged, if patients become co-infected. A study on respiratory co-infection in patients with pandemic 2009 influenza A (H1N1) virus infection showed that ICU length of stay was 3 days longer among patients who had co-infection [40].

In addition, infections and antibiotic resistance in ICU patients can also result in higher cost of treatment, and increased mortality [27]. In a study conducted by Toufen and colleagues on ICU patients in Brazil, the rate of mortality was 28.8%, while the patients with infection had a mortality rate of 34.7% and the most frequently reported infections were related to respiratory infections (58.5%) [41]*.* Chastre et al. study also suggested that the mortality rate of VAP in ICU patients varies from 20 to 50%, and even higher when caused by high-risk pathogens [42].

According to previous studies, viral-bacterial synergistic interactions are reviewed and the mortality rate can be further increased when there is simultaneous an acute respiratory viral infection and a bacterial infection. A multicenter retrospective cohort study conducted by Arabi et al. on 330 MERS SARI (Middle East Respiratory Syndrome Severe Acute Respiratory Infection) patients who were admitted to the participating ICUs showed that 18% (60 cases) and 5% (17 cases) of them had bacterial and viral co-infections, respectively [43]. It has also been estimated that one-third of the world’s population (~ 500 million people) may have been clinically infected during the 1918–19 influenza pandemic, which resulted in the death of at least 50 million people worldwide (https://www.cdc.gov/flu/pandemic-resources/1918-pandemic-h1n1.html). The findings suggest that the vast majority of individuals who died during the pandemic were infected by a bacterial infection [44].

The co-infection of the influenza virus with *Staphylococcus aureus*, especially MRSA, has been previously documented. In a study performed by Bhat et al. during the 2003–2004 influenza season, bacterial co-infections were identified in 24 of 102 cases. Accordingly, *S. aureus* was the most common etiology (11 cases); six of these 11 cases were detected as methicillin-resistant strains [45]. Randolph et al. also reported that among 838 children with influenza A (H1N1) virus admitted to a pediatric intensive care unit during the 2009 influenza A (H1N1) pandemic, 71 (8.5%) had a presumed diagnosis of early *S. aureus* co-infection of the lung with 48% positive for MRSA [46]. Moreover, Jia et al. project on mouse model findings also showed that secondary infection with *methicillin-resistant Staphylococcus aureus* after infection with influenza virus was associated with high mortality rates [47]. Another study by Liu et al. also confirmed that the co-infection of avian influenza A (H7N9) virus and extensively antibiotic-resistant *A. baumannii* in the patients with invasive mechanical ventilation is a key factor for the severity of the disease and high mortality [48].

## Conclusions

Our report is one of the first to demonstrate the presence of superinfections in the lower respiratory tract of patients with COVID-19. Our findings emphasize the concern of bacterial infections in the patients due to *A. baumannii* and *S. aureus* that are resistant to the extended-spectrum antibiotics commonly used for the treatment of life-threatening bacterial diseases, especially in ICU patients. Secondary bacterial infections may develop during or following COVID-19 and thus they are an undeniable fact. Due to severe pandemic conditions, it was not possible to have a negative control group without COVID-19 in our ICUs simultaneously. Therefore, we could not certainly state what percentages of deaths in our patients were caused by bacterial co-infections. However, when mortality rates compared to other non COVID-19 studies, e.g. 10.9% in Shiraz [29] and 17.8% in Mysuru [27], it seems that mortality is increased in COVID-19 patients and may be attributed to bacterial co-infections. Thus, further studies are recommended to confirm this finding. Mortality in COVID-19 positive patients with no underlying diseases may be due to bacterial infections that this concern also requires more investigations. Overall, it is important to limit the risk of infection and the spread of these resistant strains through controlling nosocomial infections accurately and bringing secondary infections caused by resistant bacteria that can increase the mortality rate in COVID-19 critical patients into attention.

## Data Availability

The datasets used and/or analyzed during the current study are available from the corresponding author on reasonable request.

## Abbreviations

SARS-CoV-2: Severe acute respiratory syndrome coronavirus 2
COVID-19: Coronavirus disease 2019;
2019-nCoV: 2019 novel coronavirus
NP: Nosocomial pneumonia
NI: Nosocomial infection
ICU: Intensive care unit
VAP: Ventilator-associated pneumonia
PaO_2_: Partial pressure of oxygen
FiO_2_: Fraction of inspired oxygen
CFU: Colony-forming units
CRP: C-reactive protein
RT-PCR: Reverse transcription real-time polymerase chain reaction
RNA: Ribonucleic acid
ETA: Endotracheal aspirate
AST: Antimicrobial susceptibility testing
CLSI: Clinical & laboratory standards institute
CPM: Cefepime
FOX: Cefoxitin
IMP: Imipenem
CAZ: Ceftazidime
AK: Amikacin
MEN: Meropenem
AZM: Azithromycin
GM: Gentamicin
TE: Tetracycline
CRO: Ceftriaxone
CP: Ciprofloxacin
SXT: Trimethoprim/sulfamethoxazole
LEV: Levofloxacin
P: Penicillin
E: Erythromycin
PIT: Piperacillin-tazobactam
AMS: Ampicillin-sulbactam
CTX: Cefotaxime
MIC: Minimum inhibitor concentration
ATCC: American type culture collection
MBL: Metallo-beta-lactamase
CDDT: combination disk diffusion test
MHT: modified Hodge test
MRSA: Methicillin-resistant *Staphylococcus aureus*
MSSA: Methicillin-sensitive *Staphylococcus aureus*
ESBL: Extended-spectrum beta-lactamases
